# FDA-Approved Drugs Efavirenz, Tipranavir, and Dasabuvir Inhibit Replication of Multiple Flaviviruses in Vero Cells

**DOI:** 10.3390/microorganisms8040599

**Published:** 2020-04-20

**Authors:** Michal Stefanik, James J. Valdes, Fortunatus C. Ezebuo, Jan Haviernik, Ikemefuna C. Uzochukwu, Martina Fojtikova, Jiri Salat, Ludek Eyer, Daniel Ruzek

**Affiliations:** 1 Department of Virology, Veterinary Research Institute, Hudcova 70, CZ-62100 Brno, Czech Republic; stefanik@vri.cz (M.S.); valdjj@gmail.com (J.J.V.); haviernik@vri.cz (J.H.); fojtikovamartina@seznam.cz (M.F.); salat@vri.cz (J.S.); eyer@vri.cz (L.E.); 2 Department of Chemistry and Biochemistry, Mendel University in Brno, Zemedelska 1, CZ-613 00 Brno, Czech Republic; 3 Institute of Parasitology, Biology Centre of the Czech Academy of Sciences, Branisovska 31, CZ-37005 Ceske Budejovice, Czech Republic; 4 Department of Pharmaceutical and Medicinal Chemistry, Faculty of Pharmaceutical Sciences, Nnamdi Azikiwe University, PMB 5025 Awka 420281, Nigeria; fortunatus.ezebuo@unn.edu.ng (F.C.E.); ic.uzochukwu@unizik.edu.ng (I.C.U.); 5 Faculty of Science, Masaryk University, Kamenice 5, CZ-625 00 Brno, Czech Republic

**Keywords:** FDA, flavivirus, Zika virus, tick-borne encephalitis virus, West Nile virus, antiviral

## Abstract

Vector-borne flaviviruses (VBFs) affect human health worldwide, but no approved drugs are available specifically to treat VBF-associated infections. Here, we performed in silico screening of a library of U.S. Food and Drug Administration-approved antiviral drugs for their interaction with Zika virus proteins. Twelve hit drugs were identified by the docking experiments and tested in cell-based antiviral assay systems. Efavirenz, tipranavir, and dasabuvir at micromolar concentrations were identified to inhibit all VBFs tested; i.e., two representatives of mosquito-borne flaviviruses (Zika and West Nile viruses) and one representative of flaviviruses transmitted by ticks (tick-borne encephalitis virus). The results warrant further research into these drugs, either individually or in combination, as possible pan-flavivirus inhibitors.

## 1. Introduction

The genus *Flavivirus* (family Flaviviridae) comprises more than 50 members, most of which are transmitted by mosquitoes and ticks (vector-borne flaviviruses, VBF) [1]. Despite similarities in genomic organization, replication strategy, and physicochemical properties, flaviviruses can cause a variety of diseases with clinical presentations ranging from mild fever to hemorrhagic fever, encephalitis, Guillain–Barré syndrome, and microcephaly [2]. Important human pathogens include yellow fever virus, dengue virus, West Nile virus (WNV), Zika virus (ZIKV), Japanese encephalitis virus, and tick-borne encephalitis virus (TBEV) [3,4]. No approved effective antiviral therapy directed against these viruses is currently available. To address this urgent medical need, we interrogated a library of U.S. Food and Drug Administration (FDA)-approved antiviral drugs for the ability to block flavivirus replication in vitro. Such approved drugs have well-documented modes of action, safety, and pharmacokinetic and pharmacodynamic profiles. Therefore, identifying them might expedite the regulatory process for their approval in clinical use more rapidly than new compounds [5,6,7,8,9]. 

In this study, we first performed in silico screening of a library of FDA-approved antiviral drugs for their interaction with ZIKV proteins (NS3 helicase and protease, NS5 RNA-dependent RNA polymerase, and methyltransferase). The cytotoxicities and antiviral activities of the identified hit compounds were tested against three representative flaviviruses: ZIKV and WNV as emerging mosquito-borne pathogens, and TBEV as an important tick-borne pathogen. Our results identified three FDA-approved drugs—efavirenz (an antiretroviral drug that targets the HIV-1 reverse transcriptase enzyme), tipranavir (a nonpeptidic protease inhibitor that targets the HIV protease), and dasabuvir (an inhibitor of NS5B polymerase, terminating RNA polymerization and stopping the replication of the genome of hepatitis C virus)—that inhibit flavivirus infection in vitro. To the best of our knowledge, none of these three drugs have been previously reported to have anti-VBF activity.

## 2. Materials and Methods 

### 2.1. In Silico Screen of the Library of FDA-Approved Drugs

Bioinformatics mining of the Protein Data Bank (PDB) was done to identify ZIKV proteins whose 3D structures have been deposited. The 3D atomic coordinates of six identified ZIKV protein structures (NS3 helicase (5K8T), protease (5H6V), and NS5 methyltransferase (5MRK, 5KQS, and 5ULP)) and RNA-dependent RNA polymerase (5U04) were obtained from PDB [10] and prepared for molecular docking simulation using UCSF Chimera 1.9 [11] and AutoDockTools 1.5.6 [12,13]. Briefly, all duplicate chains and hetero molecules were deleted, and polar hydrogen atoms were added. Grid box sizes, centers, and exhaustiveness were assigned to the proteins at 1.0 Å, as shown in Table 1. Respective pdbqt files were created for molecular docking simulations studies.

A library of 1960 FDA-approved drugs were obtained from Drug Bank [14] as of July 6, 2017. From this library, 73 were identified as FDA-approved antiviral drugs. These antiviral drugs were converted to their respective 3D coordinates with Open Babel 2.3.0 [15] and prepared for molecular docking simulation using AutoDockTools 1.5.6 [12,13]. Briefly, rotatable bonds were determined, all hydrogens were added, Gasteiger charges were computed, and pdbqt files were created for docking simulations studies. The prepared receptors and drugs then were used for the molecular docking simulation. 

To validate the molecular docking simulations protocol, the experimental complexes of sinefungin (SFG), 7N-methyl-8-hydroguanosine-5’-diphosphate (M7G), 5’-{[(3s)-3-amino-3-carboxypropyl][(4-fluorophenyl)methyl]amino}-5’-deoxyadenosine (KBI), 5’-guanosine-diphosphate-monothiophosphate (GSP), and (s)-2-acetamido-6-amino-n-((s)-5-guanidino-1-oxopentan-2-yl) hexanamide (7HS) with their ZIKV target proteins (NS5 methyltransferase (5MRK, 5KQS, 5ULP), NS3 helicase (5K8T), and protease (5H6V)) from PDB were reproduced in silico after ligands were obtained from the ZINC^®^ database [16] or extracted from the protein and subjected to geometry optimization. Blind docking was first performed with 5U04 and subsequently validated using M7G. AutoDockVina^®^ has a high accuracy in predicting binding free energies by setting the receptor rigid while appraising flexible ligands with a comparatively low standard error [17,18]. Therefore, receptor conformational flexibilities were neglected by rigid receptor docking. The approved drugs were docked into the receptors using AutoDockVina^®^ after the validation of molecular docking protocols. The simulations were performed locally on a Linux platform using a configuration file and script (Supplementary Material S1) containing information on the grid box centers and sizes (Table 1) of prepared receptors and selected drugs.

### 2.2. Induced Fit Simulations

The ZIKV polymerase, PDB: 5U0B without its methyltrasferase domain (residues 1–267), and the ZIKV protease, PDB: 5YOF, were used for molecular docking, as previously indicated, with an increased box size of 25_xyz_. These ZIKV structures were used since they are at higher resolution with no missing residues. The docked ZIKV enzymes were then prepared and optimized for induced fit simulations using the Schrödinger’s Maestro Protein Preparation Wizard [19]. Steric clashes were eliminated via local minimization. Note that the Maestro Protein Preparation Wizard created protonation states during optimization (default) that were used in the induced fit simulations.

The induced fit simulations were performed using the Metropolis Monte Carlo-based Protein Energy Landscape Exploration server (PELE). The PELE server is freely available at pele.bsc.es and has been previously explained [20,21]. Briefly, the PELE software executes three steps: (1) a protein/ligand local perturbation, (2) a residue side chain sampling, and (3) a global minimization. The three steps are repeated for a number of iterations within 24 h. The Metropolis Monte Carlo-based method employed by PELE accepts iterations if the enthalpy of evaporation is equal to or less than its initial value. An iteration is rejected if the enthalpy is greater than its initial value. The change in enthalpy (ΔH) is calculated by the force field known as the optimized potentials for liquid simulations (OPLS-2005) [22]. The inhibitor binding enthalpy (ΔH_L_) is calculated according to Equation (1):

(1) $$\Delta H_{L}=\Delta H_{\mathrm{ab}}-\left(\Delta H_{a}+\Delta H_{b}\right)$$

where ΔH_ab_ is the enthalpy of the entire system, including inhibitors, and ΔH_a_ and ΔH_b_ are their separate enthalpy values.

For this study, the PELE ligand refinement ready-made script was used on the re-docked apo-enzymes for initial induced fit simulations. Alterations to the ready-made script were how long the inhibitor explores a region (wait for = 5) and the region explored (spawn within = 2–7 Å). The induced fit simulations were repeated for the narrow active site and binding cavities of the ZIKV polymerase until the inhibitor approximates its coordinates within 4.5 Å. The best pose/iteration of the ZIKV polymerase simulations that approximates the coordinates was chosen for the following two steps. (1) The best pose/iteration was refined using the PELE protein motion ready-made script. (2) Multiple polymerase dockings were conducted using top representatives in an ensemble cluster (UCSF Chimera 1.9 [11]) from the protein motion simulation. For all ZIKV enzymes, pose/iteration that approximates its respective inhibitor coordinates was chosen for several rounds of induced fit simulations until the inhibitors approached < 1 Å. The modifications made on the ligand refinement ready-made script for these subsequent induced fit simulations are in Supplementary Material S2. Finally, the PELE protein motion ready-made script was used to calculate the average ΔH_L_ and positions of analogous/identical co-crystalized inhibitors and were compared with protein motion simulations of the ZIKV enzymes from the pose/iteration < 1 Å. The first 20 iterations were eliminated since the simulation reached equilibrium at this point.

### 2.3. Viruses, Cells, and Tested Compounds

The following viral strains were tested: WNV (strains Eg101, a member of genomic lineage 1 isolated from human serum in Egypt; and 13-104, a representative of genomic lineage 2 isolated from the *Culex modestus* mosquito in the Czech Republic), TBEV (strain Hypr, highly pathogenic representative of the European subtype of TBEV), and ZIKV (MR-766, a representative of the African ZIKV lineage; and Paraiba_01, a member of the Asian ZIKV lineage).

Vero cells (ATCC CCL-81, African Green Monkey, adult kidney, epithelial) were cultured in Dulbecco’s Modified Eagle Medium containing 10% fetal bovine serum, 1% L-glutamine, 100 U/mL penicillin, and 100 µg/mL streptomycin (Sigma-Aldrich, Prague, Czech Republic) at 37 °C in a 5% CO_2_ atmosphere. PS cells (porcine kidney stable) were cultured at 37 °C in Leibovitz (L-15) medium supplemented with 3% fetal bovine serum, 100 U/mL penicillin, 100 µg/mL streptomycin, and 1% L-glutamine (Sigma-Aldrich, Prague, Czech Republic). Human brain cortical astrocytes (HBCAs; ScienCell, Carlsbad, CA, USA) were cultivated at 37 °C under 5% CO_2_ atmosphere in Astrocyte medium (ScienCell, Carlsbad, CA, USA), supplemented with 6% fetal bovine serum, 100 U/mL penicillin, 100 µg/mL streptomycin (Sigma-Aldrich), and 1% astrocyte growth supplement (ScienCell, Carlsbad, CA, USA). Human neuroblastoma UKF-NB-4 cells were cultured at 37 °C and 5% CO_2_ atmosphere in Iscove’s Modified Dulbecco’s Medium, supplemented with 10% fetal bovine serum, 100 U/mL penicillin, 100 µg/mL streptomycin, and 1% L-glutamine (Sigma-Aldrich, Prague, Czech Republic). 

Paritaprevir, dolutegravir, raltegravir potassium, elvitegravir, efavirenz, and tauroursodeoxycholate sodium were obtained from Sigma-Aldrich (St. Louis, MO, USA) and delavirdine mesylate, tipranavir, dasabuvir (ABT-333), saquinavir mesylate, maraviroc, and trifluridine were obtained from ChemScene, LLC (Monmouth Junction, NJ, USA). 7-deaza-2′-*C*-methyladenosine was purchased from Carbosynth (Compton, United Kingdom). Compounds were solubilized in dimethyl sulfoxide (DMSO; 100% *v*/*v*) to make stock solution with a concentration of 10 mM.

### 2.4. Cytotoxicity Assay

For detailed cytotoxicity studies, Vero, HBCA, and UKF-NB-4 cells were seeded onto 96-well plates at a density of 10,000 cells per well and incubated for 24 h at 37 °C before being used in the experiment to form a confluent monolayer. After the incubation, tested compounds were added to the cells at concentrations of 0, 3.125, 6.25, 12.5, 25, 50, 75, and 100 µM, and treated under the same regime as during antiviral testing; i.e., pretreatment for 24 h with culture medium containing appropriate drug concentrations, then the medium was exchanged with fresh compound-containing medium. After 48 h post medium exchange, cytotoxicity was measured using the Cell Counting Kit-8 (Dojindo Molecular Technologies, Inc., Munich, Germany) according to the manufacturer’s instructions. The 50% cytotoxic concentration (CC_50_) values, representing the concentration of compound that reduced cell viability by 50%, were calculated using GraphPad Prism (version 7.04, GraphPad Software, San Diego, CA, USA) as a nonlinear regression (inhibitor vs. normalized response, variable slope). All assays were performed in three independent experiments done in triplicate.

### 2.5. Antiviral Assays

#### 2.5.1. Inhibition of ZIKV-Mediated Cytopathic Effect in a Simultaneous Treatment Assay

Twelve compounds that were found to bind with a high affinity to the selected ZIKV proteins in the in silico simulation experiment (paritaprevir, dolutegravir, raltegravir, efavirenz, elvitegravir, tipranavir, saquinavir, dasabuvir, delavirdine, maraviroc, trifluridine, and sodium tauroursodeoxycholate) were first screened for their ability to inhibit the cytopathic effect (CPE) mediated by ZIKV (strain MR-766) infection in Vero cells. In this initial screening, all compounds were tested at a single concentration of 50 µM. DMSO was added to virus-infected cells as a negative control at a concentration corresponding to a dilution of the initial drug–DMSO stock (at a maximal final concentration of 0.5% (*v*/*v*)). Culture medium containing appropriate drug concentrations and simultaneously inoculated with virus (multiplicity of infection = 0.1) was added to the cell monolayers. After 48 h of incubation at 37 °C, culture media were collected, and CPE was quantified using the Cell Counting Kit-8 (Dojindo Molecular Technologies, Inc., Munich, Germany) and expressed as percentage of cell viability. All assays were performed in three independent experiments done in triplicate.

#### 2.5.2. Anti-ZIKV Activity in a Simultaneous Treatment Assay

Compounds that reduced ZIKV-mediated CPE (>90% viability of the cells in the culture compared to the uninfected cells) were further investigated for their activity to inhibit ZIKV growth in Vero cells. The compounds were tested at a single concentration of 50 µM. DMSO was added to virus-infected cells as a negative control at a concentration corresponding to a dilution of the initial drug–DMSO stock (at a maximal final concentration of 0.5% (*v*/*v*)). Culture medium containing appropriate drug concentrations and simultaneously inoculated with virus (strain MR-766, multiplicity of infection = 0.1) was added to the cell monolayers. After 48 h of incubation at 37 °C, culture media were collected, and subjected to plaque assay as described above. All assays were performed in three independent experiments done in triplicate.

#### 2.5.3. Anti-ZIKV Activity in a Post-Treatment Assay

Dasabuvir, efavirenz, and tipranavir (i.e., drugs that inhibited ZIKV in a simultaneous treatment assay) were further used in a post-treatment antiviral study. The potency of these compounds to inhibit ZIKV 2 h post-infection was assayed in Vero cells. The cells were infected with ZIKV (strain MR-766) at multiplicity of infection = 0.1. After 2 h, the medium containing virus was removed and replaced with a fresh medium containing the tested compounds at concentration of 50 µM. DMSO was added to virus-infected cells as a negative control at a concentration corresponding to a dilution of the initial drug–DMSO stock (at a maximal final concentration of 0.5% (*v*/*v*)). After 48 h of incubation, culture media were harvested and subjected to plaque assay. 7-deaza-2′-*C*-methyladenosine was used at the same concentration and treatment regime as a reference compound. All assays were performed in three independent experiments done in triplicate.

#### 2.5.4. Anti-VBFs Activity in a Pretreatment Assay, Dose Response Study

Dose response studies of dasabuvir, efavirenz, and tipranavir were done in Vero cells infected with TBEV, WNV, and ZIKV. The antiviral effects of dasabuvir and efavirenz were evaluated at compound concentrations of 0, 12.5, 25, 30, 40, and 50 µM; tipranavir was tested at 0, 25, 40, 50, 75, and 100 µM. The cells were pretreated with tested compounds for 24 h. Then the medium was removed, and the cells were infected with the individual viruses at a multiplicity of infection of 0.1 in culture media containing appropriate drug concentration. The compound inhibitory effect was assayed against WNV (strains Eg101 and 13-104), TBEV (strain Hypr), and ZIKV (strains MR-766 and Paraiba_01). DMSO was added to virus-infected cells as a negative control at a concentration corresponding to a dilution of the initial drug–DMSO stock (at a maximal final concentration of 0.5% (*v*/*v*)). After 48 h of incubation at 37 °C, culture media were collected, and viral titer was quantified by plaque assay. All assays were performed in three independent experiments done in triplicate.

#### 2.5.5. Anti-ZIKV Activity in Human Neural Cells a Pretreatment Assay

For further characterization of the compound-mediated anti-ZIKV effect, we used UKF-NB-4 and HBCA cells, as target cells of neural origin. The cells were pretreated with serial dilutions of the drugs for 24 h. The highest drug concentration represented the highest non-toxic (>90% viability of the treated cells) concentrations for the particular cell type (for HBCA: 12.5 µM dasabuvir, 12.5 µM tipranavir, 3.125 µM efavirenz; for UKF-NB-4: 6.25 µM dasabuvir, 50 µM tipranavir, 12.5 µM efavirenz). Then the medium was removed, and the cells were infected with Paraiba_01 strain of ZIKV at a multiplicity of infection of 0.1 in culture media containing appropriate drug concentration. DMSO was added to virus-infected cells as a negative control at a concentration corresponding to a dilution of the initial drug–DMSO stock (at a maximal final concentration of 0.5% (*v*/*v*)). After 48 h of incubation at 37 °C, culture media were collected, and viral titer was quantified by plaque assay. All assays were performed in three independent experiments done in triplicate.

### 2.6. Plaque Assay

Plaque assays were performed in Vero cells (for ZIKV and WNV titers) or in the PS cells (to determine TBEV titers) as described previously [23,24,25]. The obtained viral titer values were recalculated to percentages of viral titer inhibition, applied to constructing the dose-response and inhibition curves, and used to calculate the 50% effective concentration (EC_50_). We calculated EC_50_ values using GraphPad Prism as a nonlinear regression (agonist vs. normalized response) from three independent experiments done in triplicate. 

### 2.7. Immunofluorescence Staining of Viral Antigen

The results obtained from antiviral assays were confirmed using a cell-based flavivirus immunostaining assay with a mouse monoclonal antibody that specifically recognizes the flavivirus group surface antigen, as described previously [25]. Briefly, Vero cells seeded onto 96-well plates were treated with the test compound (0, 40, or 50 μM dasabuvir or efavirenz; 0, 75, or 100 μM tipranavir) and infected with the individual viruses at a multiplicity of infection of 0.1. After incubation at 37 °C for 48 h, the cell monolayers were fixed with cold acetone-methanol (1:1), blocked with 10% fetal bovine serum, and incubated with the flavivirus antibody (1:250; Sigma-Aldrich, Prague, Czech Republic). After washing, the cells were labeled with an anti-mouse goat secondary antibody conjugated with fluorescein isothiocyanate (FITC; 1:500) and counterstained with DAPI (4′,6-diamidino-2-phenylindole; 1 μg/mL) to allow visualization of the cell nuclei. The fluorescence signal was recorded with an Olympus IX71 epifluorescence microscope and processed by ImageJ software.

## 3. Results and Discussion

**Figure 1:**
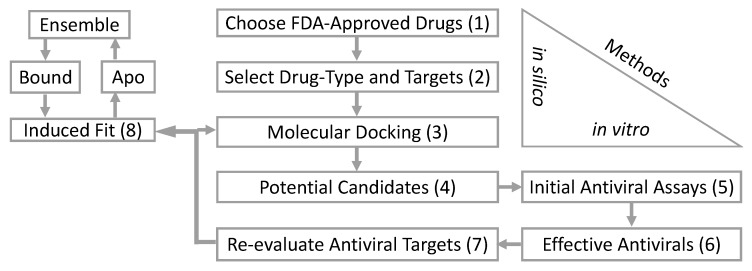
The workflow schematic indicates that a total of 1960 U.S. Food and Drug Administration (FDA)-approved drugs were downloaded from Drug Bank in the year 2017 (1). Excluding FDA-approved small molecule antiviral drugs (<900 daltons), only 73 were >1000 daltons. These 73 antivirals were then screened in silico with six Zika virus (ZIKV) proteins (2; Table 1). After the molecular docking screen (3), 12 FDA-approved antiviral drugs resulted in favorable scores (4). An in vitro assay of these 12 antivirals (5) resulted in three effective anti-ZIKV FDA-approved drugs (6). The drugs and targets were re-evaluated by searching the Protein Data Bank (PDB) for similar co-crystalized compounds (7). The molecular dockings were repeated using the average coordinates from various inhibitors. Finally, a series of induced fit simulations and dockings (8) were conducted on the favorable re-docked structures. The structures were then compared to similar co-crystalized compounds.

**Figure 2:**
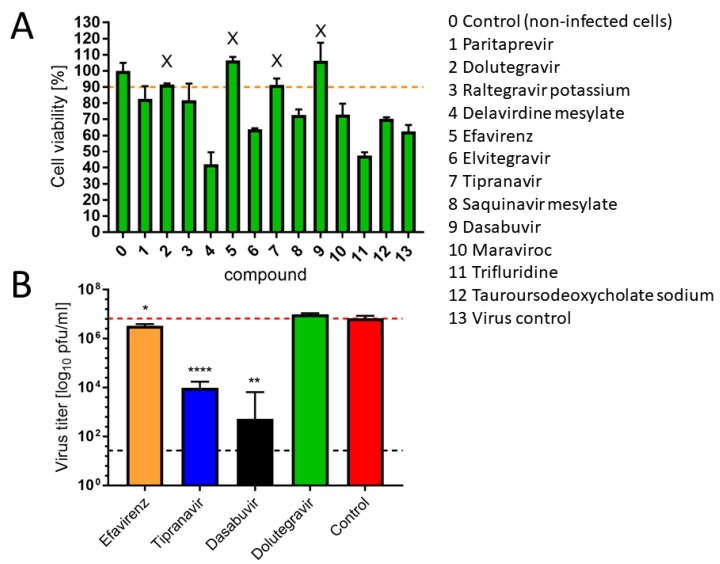
During the initial screening, all compounds identified by the molecular docking were tested at a single concentration of 50 µM for inhibition of ZIKV-mediated cytopathic effect (CPE) (simultaneous treatment assay). Dimethyl sulfoxide (DMSO) was added to virus-infected cells as a negative control and to control, non-infected cells, at a concentration corresponding to a dilution of the initial drug–DMSO stock. Culture medium containing appropriate drug concentrations and virus inoculum (multiplicity of infection = 0.1) was added to the cell monolayers. After 48 h of incubation at 37 °C, culture media were collected, and CPE was quantified using the Cell Counting Kit-8 and expressed as percentage of cell viability. The horizontal dashed line indicates 90% cell viability compared to uninfected cells (**A**). Inhibitory effect of the compounds that reduced ZIKV-mediated CPE (>90% viability of the cells in the culture compared to the uninfected cells; marked by X in (A)) on ZIKV growth, as determined by plaque assay. Horizontal dashed line indicates the minimum detectable threshold of 1.44 log_10_ pfu/mL (black), and the mean virus titer in control (untreated) cells (red). Data were analyzed by Student’s *t*-test (GraphPad Prism, version 7.04); *, *p* < 0.05; **, *p* < 0.01; ****, *p* < 0.0001 (**B**).

To date, there are no specific antivirals available for clinical use with activity against VBFs—excluding preclinical studies of small molecules with known anti-ZIKV properties [7]. To address this gap, and to identify large molecule drugs, we conducted an in silico screen of an FDA-approved library for antiviral drugs using ZIKV protein structures as a VBF-representative model (Table 1). These are the initial steps in our computational and biological workflow (Figure 1). From the in silico screening, we identified 12 of the 73 antiviral drugs with favorable docking scores. The 12 FDA-approved antiviral drugs are paritaprevir, dolutegravir, raltegravir, efavirenz, elvitegravir, tipranavir, saquinavir, dasabuvir, delavirdine, maraviroc, trifluridine, and sodium tauroursodeoxycholate. These 12 in silico selected antiviral drugs were then tested in vitro for an anti-ZIKV effect on Vero cells at a concentration of 50 µM (simultaneous treatment assay). Inhibition of ZIKV-induced CPE was monitored by light microscopy and quantified at 48 h after infection using the in vitro assay for quantitative evaluation of the cell viability, as previously described [26]. From the in vitro screening, four antivirals (efavirenz, tipranavir, dolutegravir, and dasabuvir) inhibited ZIKV-mediated CPE in cell culture (>90% viability of the infected cells compared to uninfected controls) at a concentration of 50 µM (Figure 2A). However, only three of these (efavirenz, tipranavir, and dasabuvir) reduced ZIKV titer in the culture at this concentration (Figure 2B). The antiviral effect of efavirenz, tipranavir, and dasabuvir was further demonstrated in another experiment, when these drugs were applied to ZIKV-infected Vero cells 2 h post-infection (post-treatment assay; Supplementary Figure S1). The anti-ZIKV effect of tipranavir and dasabuvir was even stronger compared to 7-deaza-2′-*C*-methyladenosine, which is known to be an effective ZIKV inhibitor with documented activity both in vitro [26] as well as in vivo [27], and was used as a reference compound in our study (Supplementary Figure S2). 

According to the initial molecular docking results, dasabuvir and tipranavir bind to the ZIKV methyltransferase (PDB: 5MRK) and efavirenz binds to ZIKV protease (PDB: 5H6V). These are apparent false positives since the non-nucleoside efavirenz and the non-peptidomimetic tipranavir have co-crystalized structures with HIV polymerase [28] and HIV protease [29], respectively. To date, there are no co-crystalized structures with dasabuvir. As a non-nucleoside inhibitor, however, dasabuvir is known to interact with hepatitis C (HCV) polymerase [30]. Given the dearth of ZIKV structures co-crystalized with non-nucleoside and non-peptidomimetic inhibitors, and the ZIKV structural diversity with HIV enzymes, the three effective ZIKV FDA-approved antivirals were therefore re-evaluated for subsequent molecular docking.

**Figure 3:**
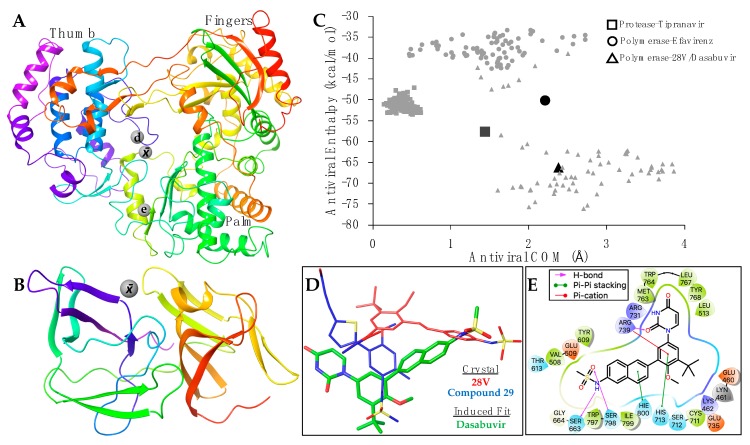
The PDB ZIKV polymerase (**A**; PDB: 5U0B) and protease (**B**; PDB: 5YOF) are color-coded from the amine-terminus (red) to the carboxyl-terminus (purple). The total average ($\bar{x}$), Dengue average (d), and HIV polymerase-efavirenz (e) binding coordinates are labeled and shown as grey spheres. The domains of ZIKV polymerase (A) are also labeled. The protein motion simulation results (**C**) show the enthalpy (y-axis) and the center of mass (COM) of the antivirals migration within its respective coordinates (x-axis). The darker/larger geometric shapes indicate average results for crystal structures of HIV protease-tipranavir (square; PDB: 6DIF), HIV polymerase-efavirenz (circle; PDB: 1FK9), and HCV polymerase-28V (triangle; PDB: 4MKB). The legend indicates the antiviral and target. The superposition (**D**) of co-crystalized antiviral structures 28V (red; PDB: 4MKB), compound 29 (blue; PDB: 5HMZ), and the best pose/iteration of ZIKV polymerase-dasabuvir (green). A ligand diagram (**E**) indicating the interacting ZIKV polymerase residues with dasabuvir and type of interaction (legend).

A PDB search resulted in 16 HCV and three Dengue polymerases co-crystalized with non-nucleoside inhibitors. Among the HCV polymerases, the non-nucleoside inhibitor 28V (PubChem ID: 46220530) is similar in composition to dasabuvir. A similar PDB search did not reveal any flavivirus proteases co-crystalized with non-peptidomimetic inhibitors. We therefore used the positions of two macrocyclic HCV protease inhibitors. The PDB accession numbers are in Supplementary Table S1. The average positions of the binding coordinates are shown with the ZIKV structures (Figure 3A,B). The ZIKV polymerase and protease, with respective inhibitors, were prepared for molecular docking as previously indicated. Conformational changes between bound and apo-enzymes at the binding site are necessary for an accurate docking pose. The top docking poses that approximate their respective average binding coordinates (Figure 3A,B) were therefore used for a series of induced fit simulations and molecular dockings—the final step of the workflow (Figure 1).

None of the PDB non-nucleoside co-crystalized with flavivirus polymerases (Supplementary Table S1) indicate that efavirenz will bind at the HIV polymerase site (Figure 3A). Molecular docking and induced fit simulations also showed that efavirenz binding at the HIV position caused large, unnatural conformational changes at the palm domain (data not shown). We therefore focused on the average polymerase binding site (Figure 3A) for efavirenz simulations. Although both efavirenz and tipranavir approach their respective binding coordinates, the enthalpy values were slightly less favorable than their co-crystalized analogs (Figure 3C). This may be due to the structural-sequence diversity between HIV and flavivirus enzymes, indicating a distinct allosteric binding site for efavirenz and tipranavir, or that these antivirals may not be as effective for ZIKV (compared with HIV). 

Of the three antivirals, however, dasabuvir clusters within the average enthalpy and coordinates of the analog 28V co-crystalized with HCV polymerase (Figure 3C). Although dasabuvir approximates the position of 28V, the coordination of dasabuvir resembles that of the non-nucleosides bound to Dengue polymerases (Figure 3A). The Dengue polymerase bound to compound 29 (PDB: 5HMZ) was used as an example (Figure 3D). Aside from the electrostatic interactions between ZIKV polymerase and dasabuvir, there are five residues that form direct contact (Figure 3E). Hydrogen bonds are formed at the methanesulfonamide group of dasabuvir with ZIKV polymerase residues Ser663 and Ser798, and Arg739 forms bonds with the dioxopyrimidine group. Pi-cation interactions are also coordinated by Arg739 along with the pi-pi stacking of His713 at the methoxyphenyl group. Lastly, His800 forms pi-pi stacking with naphthalene group of dasabuvir (Figure 3E).

We then evaluated the cytotoxic profiles and antiviral potency of efavirenz, tipranavir, and dasabuvir in detail using three cell lines: Vero cells, human neuroblastoma cells UKF-NB-4, and primary HBCAs. Both UKF-NB-4 and HBCA are target cells for neurotropic and neuroinvasive VBFs and therefore represent a clinically relevant model for our cytotoxicity/antiviral studies. In Vero cells, we observed no cytotoxicity across a concentration range 0–100 µM in the case of tipranavir (CC_50_ >100 µM; Table 2). Dasabuvir and efavirenz exerted only moderate cytotoxicity in Vero cells (CC_50_ values of 101.50 and 73.57 µM, respectively). For UKF-NB-4 cells, the highest toxicity was associated with dasabuvir (CC_50_, 21.28 µM), followed by efavirenz (CC_50_, 31.85 µM) and tipranavir (CC_50_, 89.17 µM). Of interest, the three drugs showed the highest cytotoxicity in HBCAs, with CC_50_ values of 25.98 µM for dasabuvir, 34.03 µM for tipranavir, and 16.68 µM for efavirenz (Table 3). We previously had observed a similar trend of selective toxicity (increasing toxicity as follows: Vero < UKF-NB-4 < HBCA) in our study focused on the toxicity comparison of antiviral drug arbidol in multiple cell types [31]. Our hypothesis is that the selective toxicity can be a result of different levels of drug uptake by distinct cell types and/or different levels of enzymatic conversion of the compounds into toxic metabolites. 

**Figure 4:**
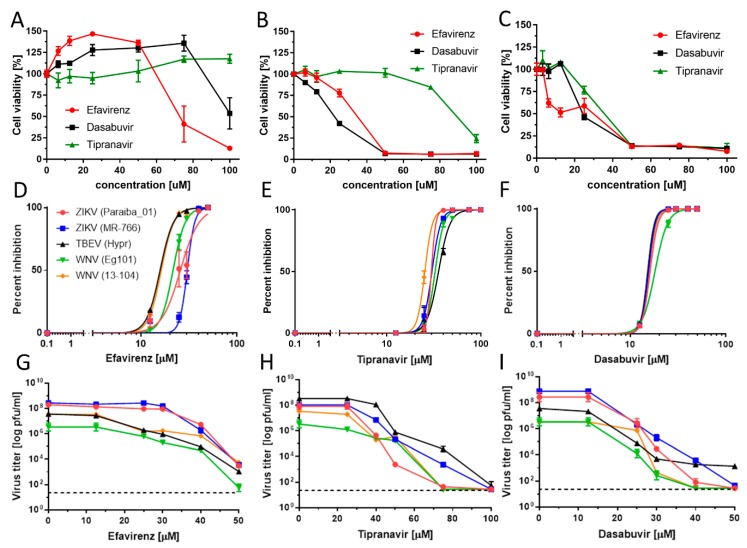
Cytotoxicity of efavirenz, tipranavir, and dasabuvir in Vero (**A**), UKF-NB-4 (**B**), and Human brain cortical astrocyte (HBCA) (**C**) cells, within the compound concentration range 0–100 μM, at 72 h post-treatment. (**D**–**F**) Virus titer inhibition curves of the indicated flaviviruses in Vero cells in the presence of serial dilutions of efavirenz (**D**), tipranavir (**E**), and dasabuvir (**F**) in a pretreatment assay. Dose-dependent effect of efavirenz (**G**), tipranavir (**H**), and dasabuvir (**I**) on virus titers in Vero cells in a pretreatment assay (legends as in **D**).

As the antiviral effect of the compounds was most pronounced using the pretreatment assays, we used this treatment regimen for all further antiviral analyses. We evaluated the antiviral effects of efavirenz, tipranavir, and dasabuvir in Vero cells against two representatives of mosquito-borne flaviviruses, ZIKV and WNV, and one representative of tick-borne flavivirus (TBEV) (pretreatment assay). All three inhibitors exhibited micromolar antiviral potency against all VBFs tested and reduced viral titers in a dose-dependent manner (Figure 4; Table 2). 

**Figure 5:**
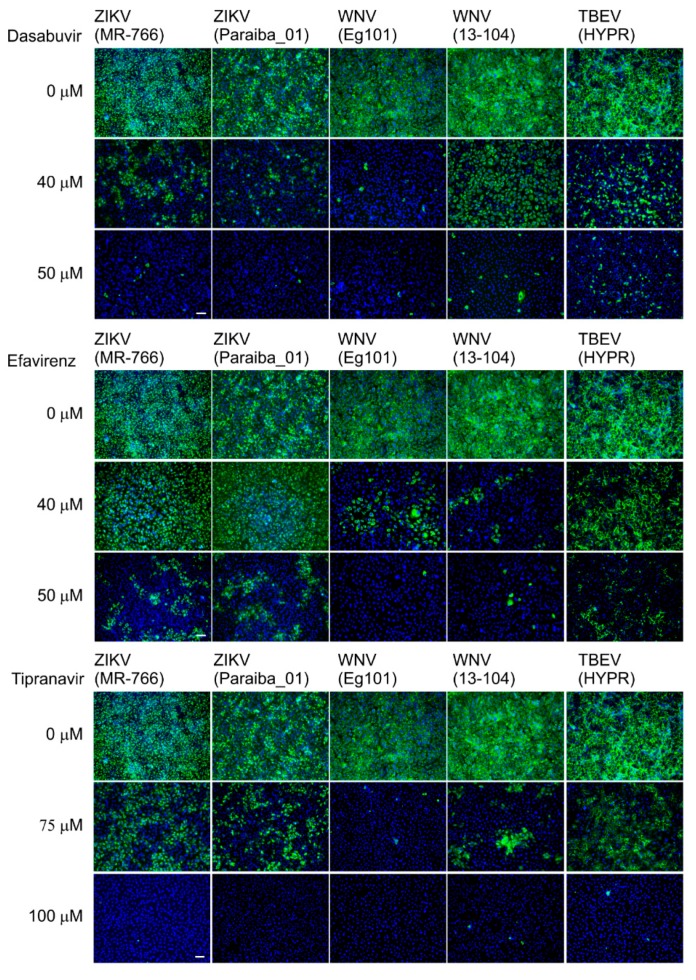
Inhibition of flaviviral surface E antigen expression by efavirenz, tipranavir, and dasabuvir. Virus-infected Vero cells were fixed on slides at 48 h p.i. and stained with flavivirus-specific antibody labeled with fluorescein isothiocyanate (FITC, green) and counterstained with 4′,6-diamidino-2-phenylindole (DAPI, blue). Scale bar, 50 μm.

Efavirenz inhibited all investigated viruses with EC_50_ values ranging from 15.86 to 30.41 µM. The strongest inhibitory effect was seen for TBEV (EC_50_, 15.86 µM) and WNV (EC_50_, 16.32 and 21.94 µM for 13-104 and Eg101, respectively). The inhibitory effect of efavirenz against ZIKV was less pronounced, with EC_50_ values of 25.78 and 30.41 µM for Paraiba_01 and MR-766, respectively. Tipranavir inhibited all VBFs tested with similar EC_50_ values, ranging from 24.17 (WNV 13-104) to 35.54 µM (TBEV). Dasabuvir showed the strongest and most robust antiviral effect in Vero cells (EC_50_ values from 15.20 (TBEV) to 18.82 µM (WNV Eg101)) (Figure 4F,I; Table 2). We further confirmed the anti-VBF activity of efavirenz, tipranavir, and dasabuvir in a cell-based flavivirus immunostaining assay, which showed a dose-dependent inhibition of surface E antigen expression by all VBFs tested in Vero cells (Figure 5).

**Figure 6:**
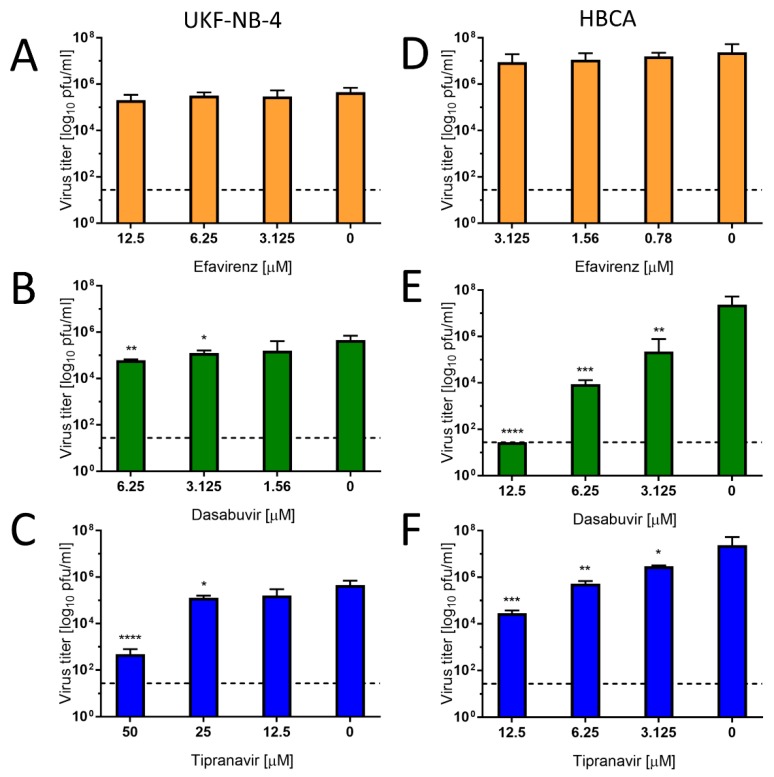
Inhibitory effect of efavirenz, tipranavir, and dasabuvir against ZIKV, strain Paraiba_01 in UKF-NB-4 (**A**,**B**, and **C**) and HBCA (**C**,**D**, and **E**) cells using a pretreatment assay. The cells were treated with serial dilutions of the drugs. The highest drug concentration represented the highest non-toxic concentrations for the particular cell type. Culture supernatants were collected at 48 h p.i., and the viral titers were determined by plaque assay. Horizontal dashed lines indicate the minimum detectable threshold of 1.44 log_10_ pfu/mL. Data were analyzed using Student’s *t*-test (GraphPad Prism, version 7.04); *, *p* < 0.05; **, *p* < 0.01; ***, *p* < 0.001; ****, *p* < 0.0001.

To investigate the antiviral effect of efavirenz, tipranavir, and dasabuvir in target cell types, primary HBCAs and human neuroblastoma cells (UKF-NB-4) were infected with Paraiba_01 strain of ZIKV and treated with serial dilutions of the drugs. At 48 h of incubation, we collected cell culture supernatants and quantified virus titer by plaque assay. In UKF-NB-4 cells, only tipranavir exhibited a significant antiviral effect against ZIKV (approx. 10^3^-fold at concentration 50 µM) (Figure 6C). The antiviral effect of dasabuvir was very low in these cells, resulting in a virus titer reduction of approximately 1 log_10_ pfu/mL at the highest concentration of the drug in comparison with mock-treated control cells (Figure 6B). No antiviral effect of efavirenz was observed in UKF-NB-4 and HBCA cells at any concentration tested (Figure 6A,D). In ZIKV-infected HBCAs, treatment with dasabuvir significantly suppressed ZIKV replication in cell culture in a dose-dependent manner; treatment with 12.5 µM of dasabuvir completely suppressed ZIKV replication in the cell culture (Figure 6E). Similarly, treatment with tipranavir significantly reduced ZIKV growth in HBCAs in a dose-dependent manner; treatment with 12.5 µM of tipranavir reduced ZIKV replication in the HBCA culture by about 10^3^-fold (Figure 6F). 

Overall, the highest and most robust antiviral effect was observed for tipranavir in human neural cells. Dasabuvir exhibited anti-ZIKV effects in astrocytes and a slight effect in neuroblastoma cells, while efavirenz had no or negligible anti-ZIKV activity in either astrocytes or neuroblastoma cells. This result indicates a cell-type-dependent activity of dasabuvir and efavirenz, which may result from differences in drug uptake or metabolic processing by different cell types. We previously observed similar cell-type-dependent activities in case of anti-VBF activities of arbidol, when the antiviral effect was found to be substantial only in HBCA and Vero cells [31], as well as for several nucleoside analogues, whose antiviral effect was studied in PS and UKF-NB-4 cells [32]. 

Repurposing of approved drugs could accelerate the development of novel therapeutic strategies, particularly for emerging life-threatening infections for which therapies are lacking [33]. In such cases, broad-spectrum antiviral drugs with effectiveness against a wide range of viral species are extremely suitable. Previous studies aimed to discover potential anti-ZIKV therapeutics via a drug-repurposing screen. The results involved different hits from the screens, and no common compounds were identified among the studies [6,7,34]. In the current work, we identified three FDA-approved drugs—tipranavir, dasabuvir, and efavirenz—that exert antiviral activities against multiple flaviviruses in vitro and that were not identified in the previous anti-ZIKV screening studies.

A sulfonamide-containing dihydropyrone tipranavir is a non-peptidomimetic protease inhibitor currently used in combination with ritonavir to treat HIV-1 infections. It is advantageous particularly in treatment-experienced patients infected with protease inhibitor–resistant HIV-1 strains [35]. Tipranavir exhibits low-nanomolar anti-HIV activity in H9 cells (EC_90_, 0.1 µM), and for multidrug-resistant HIV isolates, the EC_90_ values range from 0.31 to 0.86 µM [35,36]. To the best of our knowledge, the current work is the first study that demonstrates its activity against flaviviruses. 

Dasabuvir (previously known as ABT-333) is an inhibitor of the hepatitis C virus (HCV) and approved for use in combination with ombitasvir/paritaprevir/ritonavir for the treatment of chronic HCV infection [37,38]. In the HCV subgenomic replicon system, dasabuvir inhibits genotype 1a and 1b replicons with EC_50_ values of 7.7 and 1.8 nM, respectively [30]. In our in vitro assays, dasabuvir had a micromolar EC_50_, which was comparable to other small molecule inhibitors that showed effectiveness in laboratory animals infected with VBFs [25,26,27,39,40]. The antiviral activity of dasabuvir was demonstrated regardless if applied before infection, at the time of infection, or post-infection (Figure 2B and Figure 4, Supplementary Figure S1). The mechanism of action of dasabuvir is based on its interaction with HCV NS5B (an NS5B non-nucleoside polymerase inhibitor), leading to premature termination of synthesis of viral RNA genome [41]. Considering the structural similarities of HCV and VBF RNA-dependent RNA polymerases [42], the mechanism of action of dasabuvir could be analogous. 

Efavirenz is a non-nucleoside inhibitor that also targets HIV reverse transcriptase. In combination with other antiretroviral drugs, this agent significantly reduces HIV viral load, attenuating or preventing damage to the immune system and reducing the risk of developing AIDS [34]. The 90–95% inhibitory concentration of efavirenz for wild-type laboratory-adapted HIV strains and clinical isolates ranges from 1.7 to 25 nM when cultivated in lymphoblastoid cell lines, macrophage/monocyte cultures, and peripheral blood mononuclear cells. Efavirenz demonstrates in vitro synergistic activity against HIV-1 in combination with zidovudine, indinavir, or didanosine [43].

In summary, our present study yielded three major findings. First, our results identified three FDA-approved drugs—efavirenz, tipranavir, and dasabuvir—that inhibit replication of multiple flaviviruses in vitro in Vero cells. All three inhibitors exhibited micromolar antiviral potency against all viruses tested and reduced viral titers in a dose-dependent manner. Secondly, the anti-ZIKV effect of these drugs in Vero cells was demonstrated regardless if the compounds were applied before infection, at the time of infection, or even post-infection. Third, the anti-ZIKV effect of dasabuvir and tipranavir was confirmed also in human neural cells, which represent target cell types for the virus. One of possible limitations of our study is the fact that the mechanism of action of the identified compounds remains elusive. Future studies are also needed to investigate the anti-flavivirus effect of the identified compounds in vivo. 

## 4. Conclusions

Efavirenz, tipranavir, and dasabuvir at micromolar concentrations were identified to inhibit two representatives of mosquito-borne flaviviruses (ZIKV and WNV) and one representative of flaviviruses transmitted by ticks (TBEV). All foregoing results do not necessarily indicate that efavirenz, tipranavir, and dasabuvir are suitable candidates for treating humans infected by VBFs. These results, however, do identify novel activities. Further research on these FDA-approved antiviral drugs, either individually or in combination, will consider them as possible pan-flavivirus inhibitors. 

## Figures and Tables

**Table 1:** Grid box centers and sizes used for molecular docking simulations.

Protein	Center			Size			Exhaustiveness
	x	y	z	x	y	z	
5MRK	18.216	7.699	4.793	13	18	15	8
5K8T	115.969	2.824	64.433	14	19	14	8
5H6V	−8.522	3.178	−14.473	12	10	17	8
5KQS	52.145	10.164	−2.722	15	15	12	8
5ULP	−2.874	−1.66	26.51	15	17	16	8
5U04	25.036	68.817	103.577	12	16	16	8

Key: methyltransferases (5MRK, 5KQS, and 5ULP), NS3 helicase (5K8T), protease (5H6V), NS5 RNA-dependent RNA polymerase (5U04).

**Table 2:** Virus inhibition and cytotoxicity characteristics of efavirenz, tipranavir, and dasabuvir in Vero cells.

	Virus (Strain)	EC_50_ (µM)^a^	95% CI (µM)^b^	CC_50_ (µM)^c^	95% CI (µM)^b^	Selectivity Index (SI) (CC_50_/EC_50_)
**Efavirenz**	ZIKV (Paraiba_01)	25.78	23.54–28.03	73.57	64.27–87.43	2.85
	ZIKV (MR-766)	30.41	29.72–31.10			2.41
	TBEV (Hypr)	15.86	14.17–17.54			4.63
	WNV (Eg101)	21.94	19.58–24.30			3.35
	WNV (13-104)	16.32	14.79–17.86			4.50
**Tipranavir**	ZIKV (Paraiba_01)	29.85	25.59–34.11	134.90	130.50–143.30	4.51
	ZIKV (MR-766)	30.29	27.68–32.90			4.45
	TBEV (Hypr)	35.54	32.98–38.10			3.79
	WNV (Eg101)	32.70	30.61–34.78			4.12
	WNV (13-104)	24.17	21.07–27.27			5.58
**Dasabuvir**	ZIKV (Paraiba_01)	16.12	14.27–17.97	101.50	95.02–113.50	6.29
	ZIKV (MR-766)	15.50	11.00–19.99			6.54
	TBEV (Hypr)	15.20	8.41–21.99			6.67
	WNV (Eg101)	18.12	17.06–19.18			5.60
	WNV (13-104)	15.65	12.68–18.62			6.48

^a^ EC_50_ (50% effective concentration) values were calculated using GraphPad Prism (version 7.04, GraphPad Software, San Diego, CA, USA) as a nonlinear regression (agonist vs. normalized response) from three independent experiments done in triplicate. ^b^ 95% CI; 95% confidence interval. ^c^ CC_50_ values, representing the concentration of compound that reduced cell viability by 50%, were calculated using GraphPad Prism as a nonlinear regression (inhibitor vs. normalized response, variable slope).

**Table 3:** Cytotoxicity characteristics of efavirenz, tipranavir, and dasabuvir in UKF-NB-4 and HBCA cells.

	Cell Type	CC_50_ (µM)^a^	95% CI (µM)^b^
**Efavirenz**	UKF-NB-4	31.85	29.93–33.79
	HBCA	16.68	12.70–21.68
**Dasabuvir**	UKF-NB-4	21.28	20.13–22.48
	HBCA	25.98	22.98–29.87
**Tipranavir**	UKF-NB-4	89.17	86.90–91.46
	HBCA	34.03	30.28–38.17

^a^ CC_50_ values, representing the concentration of compound that reduced cell viability by 50%, were calculated using GraphPad Prism (version 7.04, GraphPad Software, San Diego, CA, USA) as a nonlinear regression (inhibitor vs. normalized response, variable slope). ^b^ 95% CI; 95% confidence interval.

## Supplementary Materials

The following are available online at https://www.mdpi.com/2076-2607/8/4/599/s1, Supplementary Material S1: Configuration file and script containing information on the grid box centers and sizes of prepared receptors and selected drugs. Supplementary Material S2: The modifications made on the ligand refinement ready-made script for the induced fit simulations in PELE. Supplementary Table S1: PDB accession numbers indicated by target-location. Supplementary Figure S1: Inhibitory effect of efavirenz, tipranavir, and dasabuvir on ZIKV growth in Vero cells in a post-treatment assay. Supplementary Figure S2: Inhibitory effect of a reference compound (7-deaza-2′-*C*-methyladenosine) on ZIKV growth in Vero cells.
