# Supplementary material for: FDA-Approved Drugs Efavirenz, Tipranavir, and Dasabuvir Inhibit Replication of Multiple Flaviviruses in Vero Cells
Source: Microorganisms. 2020 Apr 20;8(4):599. doi: 10.3390/microorganisms8040599 (PMC7232190; doi:10.3390/microorganisms8040599)
Supplement: Supplementary file 1 [file microorganisms-08-00599-s001.pdf]

pharmaceutics

Supplementary Material S1: Configuration file and script containing information on the grid box centers and sizes of prepared receptors and selected drugs.

Shell script

```
#! /bin/bash

for f in *.pdbqt; do
   b=`basename $f .pdbqt`
   echo Processing ligand $b
   mkdir -p $b
   vina --config conf.txt --ligand $f --out ${b}/out.pdbqt --log ${b}/log.txt
done
```

Configuration files (i.e content of conf.txt) for the different docking simulations

```
receptor	=	5h6v_only.pdbqt

center_x	=	-8.522
center_y	=	3.178
center_z	=	-14.473

size_x	=	12
size_y	=	10
size_z	=	17



receptor	=	5k8t_only.pdbqt

center_x	=	115.969
center_y	=	2.824
center_z	=	64.433

size_x	=	14
size_y	=	19
size_z	=	14
```

receptor	=	5kqs_only.pdbqt
center_x	=	52.145
center_y	=	10.164
center_z	=	-2.722
size_x	=	15
size_y	=	15
size_z	=	12

receptor	=	5mrk_only.pdbqt
center_x	=	18.216
center_y	=	7.699
center_z	=	4.793
size_x	=	13
size_y	=	18
size_z	=	15

receptor	=	5u04_only.pdbqt
center_x	=	25.036
center_y	=	68.817
center_z	=	103.577
size_x	=	12
size_y	=	16
size_z	=	16

receptor	=	5ulp_only.pdbqt
center_x	=	-2.874
center_y	=	-1.66
center_z	=	26.51

size_x	=	15
size_y	=	17
size_z	=	16

Supplementary Material S2: The modifications made on the ligand refinement ready-made script for the induced fit simulations in PELE.

```
pele &
	het Chain:ID & #The chain and identification of the drug
	pdbmodel yes &
	init_min yes &
	steric_tr 500 &
	steered 1 &
	waitfor 5 &
	task &
		show bind_ene 1 &
		spawn point 1 xyz lt n & #xyz are the specific binding coordinates; n was lowered depending on the drug position at the start of the simulation
		if random 1 gt 0.5 then tra_r 0.6 else tra_r 0.3 endif &
		if random 2 gt 0.5 then rot_r 0.25 else rot_r 0.05 endif &
		exit steps gt 100 &
	end_task &
	tries 50 &
	temp 4000 &
	anmfreq 1 &
	spfreq 1 &
	mifreq 1 &
	wrfreq 1 &
	sprad 5.0 &
	lcom_con 0.01 &
	side &
		randomize yes &
		iter 1 &
		verbose no &
		failsafe no &
	sideend &
	path traj_ &
	min &
		rmsg 0.04 &
		nbup yes &
		gbup yes &
		alphaup yes &
	minimend &
	mirad 60 &
	caconst 0.5 &
	rem_bulk_mov 3 &
```

```
anm_eig_freq 100000 &
anm_altm_freq 10 &
anm_altm_type 4 &
lanmanm neig 6 &
lanmanm mode 5 &
lanmanm move_ca 0.8 &
lanmanm mix_modes 0.80 &
lanmmin &
    mxitn 100 &
    iter 1 &
    rmsg 0.04 &
    nbup yes &
    alphaup no &
 minimend
```

Supplementary Table S1. PDB accession numbers indicated by target-location

HCV Polymerase-Active Site	Dengue Polymerase-Active Site	HCV Protease-Active site
2AWZ	5HMY	3SU3
2AX0	5HMZ	3SUD
2AX1	5HN0	
3FQK		
3FQL		
3G86		
3H5S		
3H5U		
3H59		
4MIA		
4MK7		
4MK8		
4MK9		
4MKA		
4MKB		
5W2E		

Supplementary Figure S1: Inhibitory effect of efavirenz, tipranavir, and dasabuvir on ZIKV growth in Vero cells in a post-treatment assay.

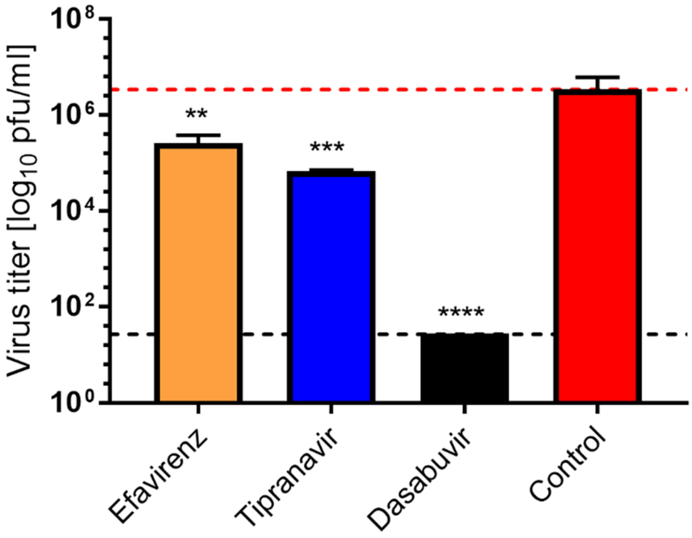

Dasabuvir, efavirenz, and tipranavir (i.e., drugs that inhibited ZIKV in a simultaneous treatment assay) were further used in a post-treatment antiviral study. The potency of these compounds to inhibit ZIKV 2 hours post-infection was assayed in Vero cells. The cells were infected with ZIKV (strain MR-766) at multiplicity of infection = 0.1. After two hours, the medium containing virus was removed and replaced with a fresh medium containing the tested compounds at concentration of 50 μM. DMSO was added to virus-infected cells as a negative control at a concentration corresponding to a dilution of the initial drug–DMSO stock (at a maximal final concentration of 0.5% (vol/vol)). After 48 h of incubation, culture media were harvested and subjected to plaque assay. Horizontal dashed black line indicates the minimum detectable threshold of 1.44 log_{10} $PFU.ml^{-1}$. Horizontal dashed red line indicates the mean titer of the control. Data were analyzed using Student's t-test (GraphPad Prism, version 7.04); **, $p < 0.01$; ***, $p < 0.001$; ****, $p < 0.0001$.

Supplementary Figure S2: Inhibitory effect of a reference compound (7-deaza-2′-C-methyladenosine) on ZIKV growth in Vero cells.

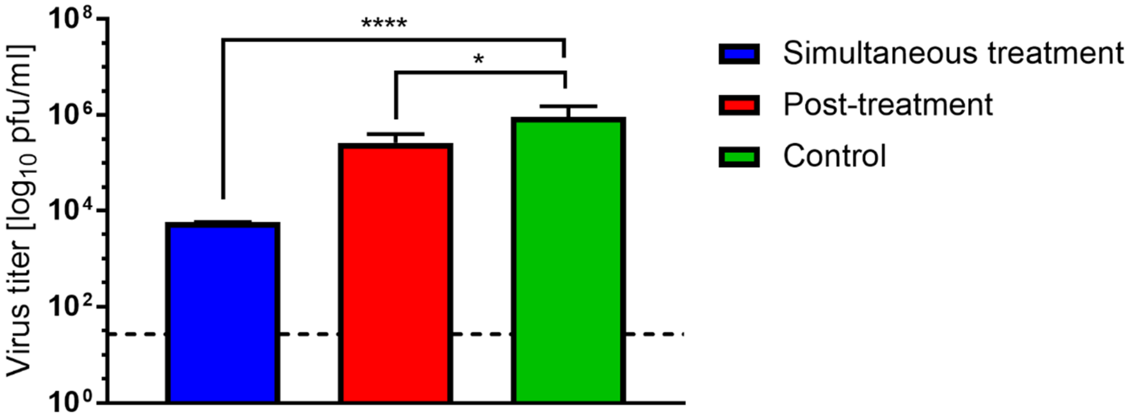

7-deaza-2′-*C*-methyladenosine was used at the concentration of 50 μM as a reference compound in our antiviral assays. The potency of 7-deaza-2′-*C*-methyladenosine to inhibit ZIKV in Vero cells was investigated in a simultaneous assay, and 2 hours post-infection assay. The cells were infected with ZIKV (strain MR-766) at multiplicity of infection = 0.1. In case of simultaneous treatment, culture medium containing 7-deaza-2′-*C*-methyladenosine at concentration of 50 μM was simultaneously added to the cell monolayers. In case of post-treatment, the medium containing virus was removed at 2 hours post-infection and replaced with a fresh medium containing 7-deaza-2′-*C*-methyladenosine at concentration of 50 μM. DMSO was added simultaneously to virus-infected cells as a negative control at a concentration corresponding to a dilution of the initial drug–DMSO stock (at a maximal final concentration of 0.5% (vol/vol)). After 48 h post-infection, culture media were harvested and subjected to plaque assay. Horizontal dashed black line indicates the minimum detectable threshold of 1.44 log_{10} PFU.ml^{-1}. Data were analyzed using Student's t-test (GraphPad Prism, version 7.04); *, $p < 0.05$; ****, $p < 0.0001$.
